# Data on expenditure, revenue, and economic growth in Nigeria

**DOI:** 10.1016/j.dib.2018.08.191

**Published:** 2018-09-05

**Authors:** Adewale F. Lukman, Olukayode Adebimpe, Clement A. Onate, Roseline O. Ogundokun, Babatunde Gbadamosi, Matthew O. Oluwayemi

**Affiliations:** a Department of Physical Sciences and Computer Science, Landmark University, Omu-Aran, Nigeria; b Department of Computer Science, Landmark University, Omu-Aran, Nigeria

**Keywords:** Expenditure, Economic growth, Revenue, Ridge parameter

## Abstract

This article describes the data for examining the influence of government expenditure and revenue on Nigerian economic growth. Data were extracted from the World Bank database and Central Bank of Nigeria (CBN) Statistical bulletin. The data are available with this article. The data is related to the research article “Newly proposed estimator for ridge parameter: an application to the Nigerian economy” (Lukman and Arowolo, 2018) but not discussed in detail. This data article will assist economists in identifying factors that will affect the economy of a country, especially in the African region.

**Specifications table**

**Table:** 

Subject area	Statistics and Economics
More specific subject area	Ridge regression; shrinkage estimators, Econometrics
Type of data	Table (Excel Format)
How data was acquired	Secondary data obtained online from the World Bank and CBN database.
Data format	Raw, filtered and analyzed
Experimental factors	The data were analyzed using the gross domestic product as a proxy for economic growth, government expenditure disaggregated into recurrent and capital expenditure, revenue disaggregated into oil and non-oil revenue.
Experimental features	Data included are collected from published data online
Data source location	Global data
Data accessibility	All the data are in this article as a supplementary file.
Related research article	[1] Lukman AF, Arowolo OT. Newly proposed estimator for ridge parameter: an application to the Nigerian economy. Pakistan Journal of Statistics. 2018 34(2):91–98.

**Value of the data**

• The data will be useful for modelling purposes, especially relating to the Nigerian economic growth.
• The data can be used to establish a relationship between capital expenditure, recurrent expenditure, and gross domestic product.
• It can also be used to examine the impact of oil and non-oil revenue on economic growth.

## 1 Data

The data consists of real gross domestic product from the World Bank database. Recurrent expenditure on economic services, Recurrent expenditure on transfers, Recurrent expenditure on social and community services, capital expenditure on economic services, capital expenditure on transfers and capital expenditure on social and community services, oil and non-oil revenue from the CBN Statistical Bulletin for Nigeria covering a period of 1970 to 2013 (see Supplementary Table 1). Real GDP is expressed in current US dollars while other variables extracted from the CBN bulletin are expressed in billion nairas.

## 2 Experimental design

### 2.1 Design

The data on the gross domestic product was obtained from the World Bank׳s World Development Indicators (WDI) [5]. The data that provides detail on government expenditure and revenue were extracted from the database of the Central Bank of Nigeria (CBN) Statistical Bulletin [6]. The gross domestic product was expressed as a function of government expenditure and revenue. The regression model is defined as follows:

(1) $$Y_{t}=\beta_{1}X_{t1}+\beta_{2}X_{t2}+\ldots +\beta_{8}X_{t8}+U_{t}$$

where $Y_{t}$ is the gross domestic product, $X_{t1}$ represent Recurrent Expenditure on Economic Services

$X_{t2}$ represent Recurrent Expenditure on Social and Community Services, $X_{t3}$ represent Recurrent Expenditure on Transfers, $X_{t4}$ represent Capital Expenditure on Economic Services, $X_{t5}$ represent Capital Expenditure on Social and Community Services, $X_{t6}$ represent Capital Expenditure on Transfers, $X_{t7}$ represent Oil Revenue and $X_{t8}$ represent Non-oil Revenue.

### 2.2 Method of data analysis

**Fig. 1:**
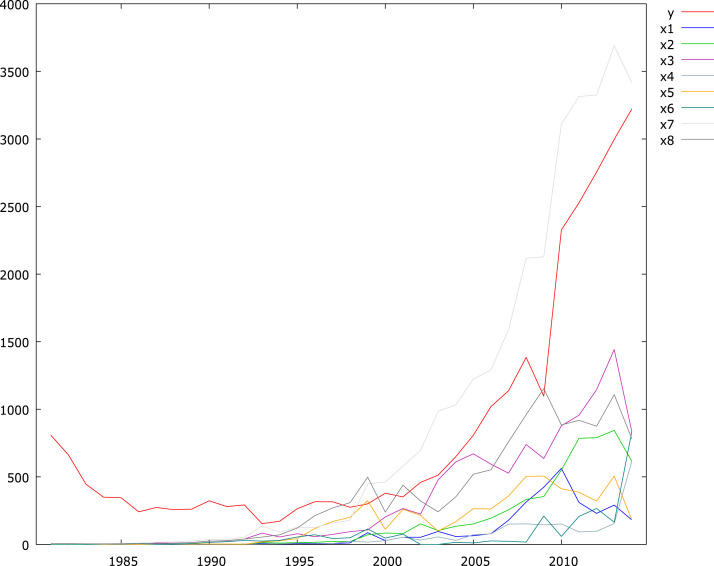
Time series plot of the dataset.

The descriptive statistics are presented in Table 1 while Fig. 1 shows the trends each of the variables follow. Table 2 provided the unit root test for the data for the original form of the data and their first difference. Cointegration test of the all the variables is provided in Table 3. The long-run estimates are provided in Table 4 using ordinary least squares (OLS). Articles [1], [2], [3], [4] suggested the use of a ridge estimator as an alternative to OLS. Readers can access article [1], [2], [3], [4] for further details. The ridge regression estimate is also provided in Table 4.

**Table 1:** Descriptive statistics of government expenditure, revenue and economic growth data.

**Statistics**	**y**	**x1**	**x2**	**x3**	**x4**	**x5**	**x6**	**x7**	**x8**
Mean	6.295	2.416	2.995	4.482	2.464	3.639	2.859	5.198	4.685
Median	5.900	2.248	3.079	4.487	2.534	4.742	3.199	5.124	5.483
Maximum	8.078	6.333	6.738	7.274	6.422	6.226	6.727	8.213	7.050
Minimum	5.035	−1.772	−1.238	1.221	−1.437	−0.421	−4.483	1.558	1.411
Std.dev	0.868	2.662	2.749	1.995	2.106	2.325	2.171	2.272	1.971
Skewness	0.771	−0.172	−0.264	−0.281	−0.034	−0.430	−1.142	−0.236	−0.429
Kurtosis	2.431	1.669	1.694	1.721	1.746	1.506	5.275	1.687	1.628
Jarque–Bera(*P*-value)	3.825 (0.148)	2.679 (0.262)	2.809 (0.245)	2.762 (0.251)	2.233 (0.327)	4.208 (0.122)	14.294 (0.000)	2.759 (0.252)	3.710 (0.156)

**Table 2:** Unit root test of the dataset.

**Variable**	**Statistics**	**Intercept**	**Intercept and trend**
$Y_{t}$	Value	0.4291	−2.3519
	*P*-value	(0.9813)	(0.3963)
$\Delta Y_{t}$	Value	−5.3024	−6.6646
	*P-*value	(0.0001)	(0.0000)
$X_{1t}$	Value	−1.1737	−2.5399
	*P-*value	(0.6739)	(0.3083)
$\Delta X_{1t}$	Value	−6.8465	−6.8692
	*P-*value	(0.0000)	(0.0000)
$X_{2t}$	Value	−1.4312	−3.5514
	*P-*value	(0.5533)	(0.0502)
$\Delta X_{2t}$	Value	−4.6616	−4.8760
	*P-*value	(0.0009)	(0.0026)
$X_{3t}$	Value	−1.2654	−1.6835
	*P-*value	(0.6336)	(0.7360)
$\Delta X_{3t}$	Value	−7.1575	−7.2801
	*P-*value	(0.0000)	(0.0000)
$X_{4t}$	Value	−0.1080	−4.4655
	*P-*value	(0.9404)	(0.0061)
$\Delta X_{4t}$	Value	−8.3017	−8.3030
	*P-*value	(0.0000)	(0.0000)
$X_{5t}$	Value	−0.9523	−1.5575
	*P-*value	(0.7583)	(0.7880)
$\Delta X_{5t}$	Value	−5.9018	−5.8174
	*P-*value	(0.0000)	(0.0000)
$X_{6t}$	Value	−4.1788	−2.3917
	*P-*value	(0.0027)	(0.3752)
$\Delta X_{6t}$	Value	−4.9088	−4.8914
	*P-*value	(0.0006)	(0.0032)
$X_{7t}$	Value	−1.2166	−1.8630
	*P-*value	(0.6549)	(0.6507)
$\Delta X_{7t}$	Value	−7.8282	−7.9323
	*P-*value	(0.0000)	(0.0000)
$X_{8t}$	Value	−1.0269	−1.0042
	*P-*value	(0.7320)	(0.9297)
$\Delta X_{8t}$	Value	−5.7478	−5.7949
	*P-*value	(0.0000)	(0.0000)

**Table 3:** Cointegration test of the dataset.

Hypothesized No. of CE(s)	Eigenvalue	Trace statistic	0.05 Critical value	Prob.**
None*	0.961076	345.9040	197.3709	0.0000
Atmost 1*	0.918395	251.7656	159.5297	0.0000
Atmost 2*	0.859054	179.0955	125.6154	0.0000
Atmost 3*	0.795576	122.2736	95.75366	0.0002
Atmost 4*	0.622168	76.23442	69.81889	0.0140
Atmost 5*	0.572335	48.00854	47.85613	0.0484
Atmost 6*	0.412339	23.37552	29.79707	0.2281
Atmost 7*	0.226481	7.958979	15.49471	0.4698
Atmost 8*	0.017488	0.511646	3.841466	0.4744

* Significance at 10%.

** Significance at 5%.

**Table 4:** Long Run Estimates of government expenditure and revenue on economic growth.

**Ordinary least squares estimator**					**Ridge estimator**	
**Regressors**	**Coefficient**	**Std.error**	**t-stat**	**VIF**	**Regressors**	**Coefficient**
const	4.185	2.03318	2.058		const	7.5728
$X_{1t}$	−0.411	0.371829	−1.104*	98.101	$X_{1t}$	0.4938
$X_{2t}$	−0.203	0.315069	−0.6454	74.622	$X_{2t}$	−0.3482
$X_{3t}$	−1.073	0.758466	−1.415	230.034	$X_{3t}$	0.829
$X_{4t}$	0.519	0.205811	2.523**	18.863	$X_{4t}$	0.293
$X_{5t}$	0.054	0.261852	0.2069	36.848	$X_{5t}$	0.138
$X_{6t}$	0.037	0.0799120	0.4606	2.953	$X_{6t}$	−0.044
$X_{7t}$	1.991	1.21072	1.645	757.146	$X_{7t}$	−1.455
$X_{8t}$	−0.724	0.479498	−1.510	89.515	$X_{8t}$	0.139
Jarque−Bera test of normality	1.260 (0.5327)				*k*	0.0033

* Significance at 10%.

** Significance at 5%.
