# Supplementary material for: Data on expenditure, revenue, and economic growth in Nigeria
Source: Data Brief. 2018 Sep 5;20:1704–9. doi: 10.1016/j.dib.2018.08.191 (PMC6157293; doi:10.1016/j.dib.2018.08.191)
Supplement: Supplementary file 1 — Supplementary material [file mmc1.docx]

DATA ON EXAMINING THE INFLUENCE OF ENERGY CONSUMPTION, CARBON EMISSIONS ON ECONOMIC GROWTH

^1*^Adewale F. Lukman, ^1^Adebimpe Olukayode, ^1^Clement A. Onate, ^1^Ogundokun O. Roseline,

^1^ Department of Physical Sciences and Computer Science, Landmark University, Omu-Aran, Nigeria.

Email: adewale.folaranmi@lmu.edu.ng, adebimpe.olukayode@lmu.edu.ng, onate.clement@lmu.edu.ng, ogundokun.roseline@lmu.edu.ng

**Conflict of Interest Form**

There is no conflict of interest with any individuals or agents.
